# Origin of the *Aromatic* Group of Cultivated Rice (*Oryza sativa* L.) Traced to the Indian Subcontinent

**DOI:** 10.1093/gbe/evz039

**Published:** 2019-02-21

**Authors:** Peter Civáň, Sajid Ali, Riza Batista-Navarro, Konstantina Drosou, Chioma Ihejieto, Debarati Chakraborty, Avik Ray, Pierre Gladieux, Terence A Brown

**Affiliations:** 1 Manchester Institute of Biotechnology, School of Earth and Environmental Sciences, University of Manchester, United Kingdom; 2 INRA-Université Clermont-Auvergne, UMR 1095 GDEC, Clermont-Ferrand, France; 3 Institute of Biotechnology & Genetic Engineering, The University of Agriculture, Peshawar, Pakistan; 4 School of Computer Science, University of Manchester, United Kingdom; 5 Department of Molecular Biology and Biotechnology, University of Kalyani, India; 6 Center for Studies in Ethnobiology, Biodiversity, and Sustainability (CEiBa), Malda, West Bengal, India; 7 BGPI, Université de Montpellier, INRA, CIRAD, Montpellier SupAgro, Montpellier, France

**Keywords:** population genomics, domestication, local ancestry inference, chloroplast genome

## Abstract

The *aromatic* group of Asian cultivated rice is a distinct population with considerable genetic diversity on the Indian subcontinent and includes the popular Basmati types characterized by pleasant fragrance. Genetic and phenotypic associations with other cultivated groups are ambiguous, obscuring the origin of the *aromatic* population. From analysis of genome-wide diversity among over 1,000 wild and cultivated rice accessions, we show that *aromatic* rice originated in the Indian subcontinent from hybridization between a local wild population and examples of domesticated *japonica* that had spread to the region from their own center of origin in East Asia. Most present-day *aromatic* accessions have inherited their cytoplasm along with 29–47% of their nuclear genome from the local Indian rice. We infer that the admixture occurred 4,000–2,400 years ago, soon after *japonica* rice reached the region. We identify *aus* as the original crop of the Indian subcontinent, *indica* and *japonica* as later arrivals, and *aromatic* a specific product of local agriculture. These results prompt a reappraisal of our understanding of the emergence and development of rice agriculture in the Indian subcontinent.

## Introduction

Rice (*Oryza sativa* L.) is one of the oldest and globally most important staple crops. The tradition of rice cultivation spans several millennia, a multitude of cultures and diverse ecogeographic regions from Iran, across the Indian subcontinent, to East and Southeast Asia. Phenotypic diversity found in rice cultivar groups and traditional landraces mirrors this richness of agroecological settings and the complex population histories. Two major groups of rice—Hsien and Keng—have been recognized in China at least since the Han dynasty (∼1,800 years B.P.) (Oka 1988), corresponding to the subsp. *indica* and subsp. *japonica*, respectively. Generally, these two groups can be distinguished by their grain shape and amylose content (the *indica* grain being usually longer; the *japonica* grain having low amylose content, making it sticky after cooking) and some agroecological characteristics (e.g., cold tolerance typical for *japonica*) (Oka 1988). Genetically, the *indica* and *japonica* groups are well-differentiated; however, several additional groups can also be recognized (Glaszmann 1987; Garris et al. 2005; Zhao et al. 2011). Besides a further subdivision of *japonica* into tropical and temperate ecotypes, two other genetically distinct groups are cultivated extensively. These are *aus* and *aromatic*, first differentiated in population genetic terms by Glaszmann (1987), who designated them as “group II” and “group V,” respectively. Genetic distinctiveness of the *aromatic* group is further documented by limited cross-compatibility with *indica* and *japonica* (Engle et al. 1969), possibly driven by cytoplasm–nucleus interactions (Virmani et al. 1981).

Recent findings suggest that the *indica*, *japonica*, and *aus* groups originated from three different gene pools of wild *Oryza rufipogon* Griff., although the degree to which the different types were domesticated independently or arose as a result of gene flow between cultivated populations and/or wild rice remain controversial (Huang et al. 2012; Civáň et al. 2015; Choi et al. 2017; Civáň and Brown 2017, 2018). In contrast, less attention has been directed at the origin of *aromatic* rice. This is probably due to the apparent genetic proximity of the *aromatic* and *japonica* types (Zhao et al. 2011; Wang et al. 2018) and the implicit assumption that *aromatic* is a subtype of *japonica*. Nonetheless, the population history of the *aromatic* group appears to be more complex than a simple postdomestication divergence from *japonica*. The first observation hinting at this complexity is a discrepancy in geographic distribution. While *japonica* rice originated in Southeast China (Huang et al. 2012; Civáň et al. 2015; Civáň and Brown 2018) and is a traditional crop across East and Southeast Asia, *aromatic* rice has its center of diversity along the Himalayan foothills and is traditionally grown in Pakistan and India, with its easternmost occurrence in Myanmar (Glaszmann 1987; Khush 2000). Secondly, the pattern of genetic diversity displayed by *aromatic* rice seems to indicate affiliation with the *aus* group. This is indicated by the main axes of genetic variation (Zhao et al. 2011), and also by analysis of 31 selective sweep regions colocated in all groups of cultivated rice (Civáň et al. 2015).

The earliest written record of rice that likely belonged to the *aromatic* group is from the Sanskrit text Susruta Samhita dated around 400 BC (Singh 2000). The *aromatic* group owes its name to the presence of fragrance in the grain, which is one of the most valued characteristics of rice. When combined with other quality traits (e.g., amylose content, grain length, grain elongation after cooking, texture of cooked rice), fragrant varieties are considered superior and have high cultural significance in many regions of Asia (Singh 2000). In most cases, enhanced fragrance is caused by BADH2 protein deficiency resulting from an 8-bp deletion that introduces a premature stop codon in the seventh exon of the *Badh2* gene (the *badh2.1* allele) (Bradbury et al. 2008; Chen S et al. 2008).

Although group V, according to Glaszmann’s classification (Glaszmann 1987), is “*aromatic*” rice, the presence of aroma or fragrance is neither a universal nor an exclusive trait of this group. Many group V varieties lack fragrance and the *badh2.1* allele frequency in this population was estimated at 0.6 (Kovach et al. 2009). Moreover, fragrant varieties can be found among *indica* (most notably Jasmine rice) and tropical *japonica*, although at much lower frequencies (Kovach et al. 2009). Since the “group V” designation has not been universally accepted, recent publications inconsistently describe this group as *aromatic* (Zhao et al. 2011; Civáň et al. 2015), Basmati/Sadri (3,000 Rice Genomes Project 2014), or circum-Basmati (Wang et al. 2018). The “Basmati” designations refer to the most produced and commercially successful varietal group, and do not fully appreciate the genetic diversity present in group V. Because it is practical to identify group V as an infraspecific taxonomic rank of *O. sativa* (as subspecies, varietas, or forma), the group name should follow the code of nomenclature and the conventions for infraspecific epithets (Turland et al. 2018). Here, we use the epithet “*aromatic*” (italicized) when referring to the whole group V population irrespective of fragrance, and the term “fragrant rice” when referring to the aroma trait.

In this article, we unravel the genomic history of the *aromatic* population by analyzing a unique genome-wide *sativa-rufipogon* diversity data set. We identify the source populations and present the most parsimonious explanation for the origin of *aromatic* rice. In doing so, we bring a new perspective on the origins of rice agriculture on the Indian subcontinent.

## Materials and Methods

### Remapping of the Wild Data Set

Raw data for 461 wild rice accessions (Huang et al. 2012) were obtained from the European Nucleotide Archive database. Read mapping and SNP-calling was carried out using the pipeline implemented in TOGGLE v0.3.3 (Monat et al. 2015). Paired-reads were mapped to the IRGSP-1.0 Nipponbare reference genome using the *aln* and *sampe* commands in BWA (Li and Durbin 2009). Alignments were sorted with *picardToolsSortSam* (http://broadinstitute.github.io/picard/) and *samToolsView*. The tool *realignertargetcreator* was used to define intervals to target for local realignment and *indelrealigner* to perform local realignment of reads around indels, both in the GATK package (McKenna et al. 2010). *Markduplicates* from picardtools was used to remove duplicates. Output bam files were divided into per chromosome bam files with bamtools. SNPs were called for each chromosome with *GATK haplotypecaller* (McKenna et al. 2010) using the *badcigar* filter. High-confidence SNPs were identified using GATK *variantfiltration* with the parameters DP > 10 and QUAL > 30. The per-chromosome vcf files were then combined into a single vcf file.

### Construction of the Merged SNP Matrix

The LD pruned 404k CoreSNP data set was downloaded from http://oryzasnp-atcg-irri-org.s3-website-ap-southeast-1.amazonaws.com/ (last accessed October 2017) and converted to ped format using PLINK v1.90 (Chang et al. 2015; www.cog-genomics.org/plink/1.9/). PCA was performed with smartpca (Patterson et al. 2006), using the *lsqproject* option without outlier removal. This sample set contained a large number of intermediate and apparently misclassified accessions (supplementary fig. S1, Supplementary Material online). To extract “typical” representatives of *indica*, *japonica*, *aus*, and *aromatic* (instead of maximizing the diversity within those groups), the medians for the top ten PCs in each group were identified and those accessions falling within 1 SD around the median in all cases extracted. This procedure produced a subset of 283 *indica*, 154 *japonica*, 124 *aus*, and 34 *aromatic* accessions (only accessions where reclassification was not needed were kept; supplementary table S1, Supplementary Material online). This subset was then extracted from the 18 million Base SNP data set that had been created by the 3k RGP by removing SNPs with excessive heterozygous calls from the complete biallelic SNP data set (http://oryzasnp-atcg-irri-org.s3-website-ap-southeast-1.amazonaws.com/; last accessed October 2017), and converted to vcf format with PLINK v1.90. From this 3k RGP subset and the newly created wild SNP matrix, all biallelic sites with identical position, reference, and alternative identifiers were extracted (with *bash* pipelines), creating compatible subsets. We noted that the reference variants of the 3k RGP data set did not always match the IRGSP-1.0 reference. We corrected those positions accordingly in a separate file (swapping the REF and ALT columns together with the called variants, using *bash* pipelines), added them to the compatible subset, and merged the two compatible subsets with GATK combinevariants command. Finally, all polymorphic sites that had at least one nonmissing data point in the wild subset were extracted, yielding a vcf file containing ∼2.4 million positions scored for 595 cultivated and 461 wild rice accessions.

### Analyses of the Merged Data Set

PCAs were performed as above; however, due to the high proportion of missing data in the wild subset, the axes of variation were calculated using the domesticated accessions only, with subsequent mapping of the wild accessions onto these axes. Allelic frequencies, nucleotide diversity and pairwise *F*_ST_ scans were calculated with VCFtools 0.1.15 (Danecek et al. 2011). Neighbor-Net networks (Bryant and Moulton 2004) were constructed in SplitsTree4, using 1–ibs (identity-by-state) distance matrix calculated in PLINK v1.9. The joint allele frequency spectrum was calculated from a table of allelic frequencies in LibreOffice Calc, using basic operations together with the functions FREQUENCY, LOG10, and conditional formatting. For the purpose of the individual-based tracking of the non-*japonica* genomic fraction in *aromatic*, *aus*, and *indica*, only major alleles (group frequency >0.5) with <0.01 frequency in *japonica* were considered. Sites with >1/3 missing data per group were ignored, since the major variant at those sites can be a deletion. A complete set of such alleles was identified from a table of allelic frequencies, extracted from the vcf file, and proportions of sites matching the group’s major allele was calculated for each individual in a spreadsheet.

### Reconstruction of the Complete Chloroplast Genomes

This part of our work was previously posted, with additional details, on a preprint server (Civáň and Brown 2016). Raw sequencing data for 508 *indica*, 482 *japonica*, and 460 wild accessions (ERP001143, ERP000729, ERP000106) (Huang et al. 2012) plus 124 *aus* and 34 *aromatic* accessions selected from the 3,000 Rice Genomes Project (2014) (ERP005654) were downloaded from the Sequence Read Archive (http://ncbi.nlm.nih.gov/Traces/sra/) using the fastq-dump command from sratoolkit 2.3.5 (additional details in supplementary table S2, Supplementary Material online). For each of the 1,608 accessions, reads significantly matching known rice chloroplast genomes (*E* value <1e-5) were extracted using the filter_by_blast script from seq_crumbs-0.1.9. Adapter contamination and low-quality regions were removed from the matching reads by Trimmomatic-0.33 (Bolger et al. 2014). The filtered and trimmed data sets were imported into Geneious 6.1 (http://www.geneious.com) and individually mapped onto the Nipponbare chloroplast genome (KM088016) used as a reference (5 mapping iterations; maximum of 5% mismatches and 10% gaps per read; maximum gap size set to 100; index word length 13; only paired reads matching within the expected distance used). The second copy of the inverted repeat region (the duplicated fraction of the chloroplast genome) was removed and the sequences were aligned in Geneious. Treating gaps as missing data, all SNPs with frequencies >0.005 were extracted, yielding a data matrix with 215 positions and 0.6% missing data points, and a median-joining network was built in Network 4 (Bandelt et al. 1999).

### Local Ancestry Inference

Phased haplotypes were imputed from the merged data set separately for the *aromatic* group and its source populations using Beagle 5.0 (Browning and Browning 2007) with default parameters. We noticed that changing the *ne* parameter by orders of magnitude does not have an obvious impact on the subsequent local ancestry inference. However, the sample size of the putative source populations does; therefore, we randomly selected 30 *japonica* accessions to match the size of the non-*japonica* source sample (supplementary table S1, Supplementary Material online). Using these two samples as the putative source populations, the local ancestry of the *aromatic* haplotypes was inferred with Loter (Dias-Alves et al. 2018). Loter does not require specifications of uncertain biological parameters (genetic maps; recombination and mutation rate; average ancestry coefficients; number of generations since admixture) and has been shown to outperform other tools for local ancestry inference of ancient admixture events (Dias-Alves et al. 2018). Nonetheless, we tested the inference accuracy on our data. Seven *aus* individuals from the non-*japonica* source population were recombined in silico with seven randomly chosen *japonica* individuals (supplementary table S1, Supplementary Material online), using one recombination point per chromosome to split each chromosome into two equal ancestry blocks. These 14 in silico recombinants were phased and their local ancestry was inferred as above.

## Results

### Merged Data Set of Nucleotide Diversity

**Fig. 1.**
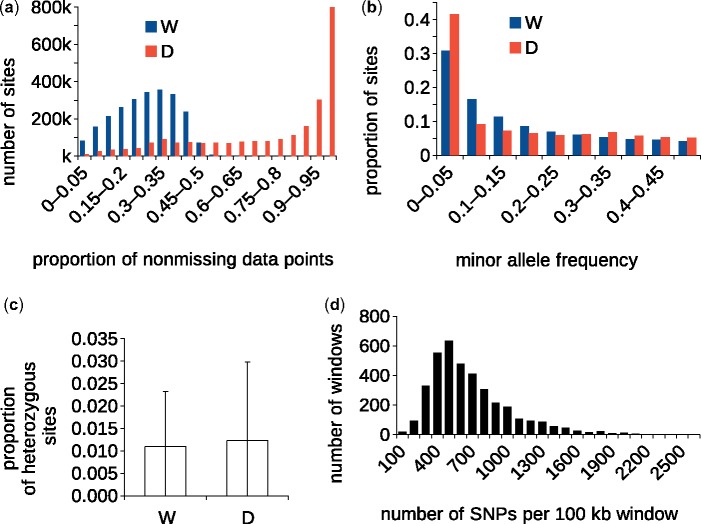
—Descriptive statistics of the merged SNP data set. (*a*) Histogram of nonmissing data (per site) for the two superpopulations in the merged data set—wild (W) and domesticated (D). (*b*) Frequency distribution of major alleles in the wild (W) and domesticated (D) superpopulations. (*c*) Mean observed heterozygosity for the wild (W) and domesticated (D) superpopulations. Error bars indicate SD. (*d*) Histogram of SNP densities in the merged data set, across the entire genome.

Recently, 76 *aromatic* genomes have been sequenced on the Illumina platform in depths sufficient for reference-based genome reconstruction. The resulting diversity data are publicly available within the 3,000 Rice Genome Project data set (a.k.a. 3k RGP) (3,000 Rice Genomes Project 2014; Wang et al. 2018). However, the 3k RGP data set does not contain data from *O. rufipogon*—the wild progenitor of cultivated rice—and is not directly compatible with previous data sets of wild diversity due to different reference sequences being used (IRGSP4 in Huang et al. 2012 and IRGSP-1.0 in 3,000 Rice Genomes Project 2014). We therefore remapped the wild rice data of Huang et al. (2012) onto the newest Nipponbare reference (IRGSP-1.0) and created a new single nucleotide polymorphism (SNP) data set by merging the mapped wild data with a subset of the 3k RGP data. This new IRGSP-1.0-based genome-wide nucleotide diversity matrix comprises 595 domesticated accessions (283 *indica*, 154 tropical and temperate *japonica*, 124 *aus*, 34 *aromatic*) (3,000 Rice Genomes Project 2014) and 461 wild rice accessions (Huang et al. 2012) and consists of 2,365,188 biallelic positions. The domesticated and wild subsets are heterogeneous in respect to the proportion of scored data points (fig. 1*a*), which can be mainly attributed to different sequencing depths in the two subsets. While the wild subset was constructed from data with mean sequencing depth ∼2×, the mean mapping depth of the 3k RGP subset is 14× (7.5× and 27.2× for 5th and 95th percentile, respectively), indicating that the missingness in the latter group is to a large extent caused by presence–absence variation (i.e., indels). Per individual proportions of missing data vary between 0.32–0.97 in wild rice and 0.07–0.54 in domesticated rice. The distribution of allelic frequencies in cultivated rice is markedly skewed toward the extreme categories, with 42% of variants approaching fixation (minor allelic frequency ≤0.05), compared with 31% in wild rice (fig. 1*b*). This difference is likely to be a consequence of the domestication bottleneck, selection, and crop homogenization, facilitated by the predominantly self-pollinating mode of reproduction in *O. sativa*. The mean proportion of heterozygous sites is not significantly different in wild and domesticated rice (fig. 1*c*), although this observation is probably biased by the low sequencing depths in wild rice (the proportion of heterozygous sites in wild rice is expected to be higher due to more frequent cross-pollination; Ishii et al. 2013). The SNPs are distributed across all twelve rice chromosomes, with average density of 6.3 SNP/kb (supplementary fig. S2, Supplementary Material online) and a modal category 400–500 SNPs per 100-kb window (fig. 1*d*).

### Population Structure and Genome-Wide Diversity Scans

**Fig. 2.**
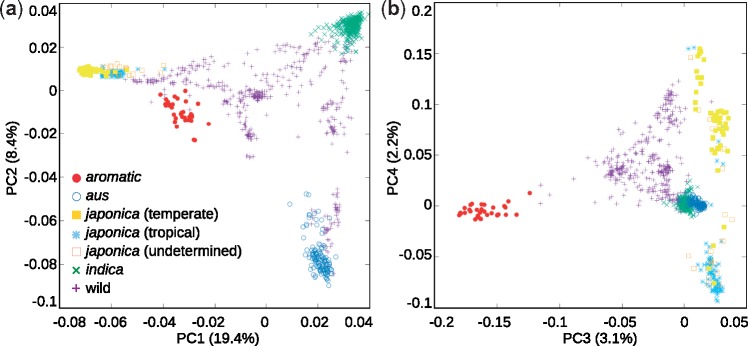
—PCA computed from the ∼2.4 million bialleleic sites in the merged data set. (*a*) The top two axes of variation jointly explaining 27.8% of the total variation. (*b*) The third and fourth axes jointly explaining 5.3% of the total variation.

Principal component analysis (PCA) of the 2,365,188 polymorphic sites separates *O. sativa* accessions into five statistically strong groups: tropical *japonica*, temperate *japonica*, *indica*, *aus*, and *aromatic*. Analysis of variance shows that the PC values of each of these five groups are distinct with high statistical significance (*P* value <1e-6) along the first four PCs jointly explaining 33% of the total variation, with the exception that tropical+temperate *japonica* are not distinguished along PC3, and *indica*+*aus* are not distinguished along PC4 (fig. 2*a* and *b*). The diversity of the *aromatic* group is clearly defined and separated from other cultivated groups along PCs1–3. Nonetheless, the position of the *aromatic* cluster along the first two PCs suggests ambiguous relationships to *japonica* and *aus*, and there is a strong overlap between *aromatic* and the *indica+aus* clusters along PC4. We further investigated these relationships through diversity and pairwise *F*_ST_ scans across the entire genome. These scans confirmed that parts of the *aromatic* genome have near-zero pairwise *F*_ST_ with *japonica*, these parts often coinciding with regions of low diversity in *aromatic* (supplementary fig. S3*a* and *b*, Supplementary Material online). Such observations suggest that parts of the *aromatic* genome are shared with *japonica*, and that some of those segments were subjected to selection in *aromatic*. However, a similar relationship between *aromatic* and the other cultivated groups is not apparent in the pairwise *F*_ST_ scans (supplementary fig. S2*c* and *d*, Supplementary Material online), providing no indications for *japonica*×*aus* or *japonica*×*indica* hybridization in the ancestry of *aromatic*.

The Neighbor-Net network clearly separates *indica*, *japonica*, *aus*, and *aromatic* into four clusters in the context of wild diversity (supplementary fig. S4, Supplementary Material online). High network complexity (1,056 accessions; 214,146 edges) obscures the origins of *aromatic*, which become clearer after exclusion of the wild populations (supplementary fig. S5, Supplementary Material online). This second network shows that *aromatic* shares unique edges with *japonica*, separating these two groups from the rest, but also with *aus*, separating *aus* and *aromatic* from *japonica* and *indica*.

**Fig. 3.**
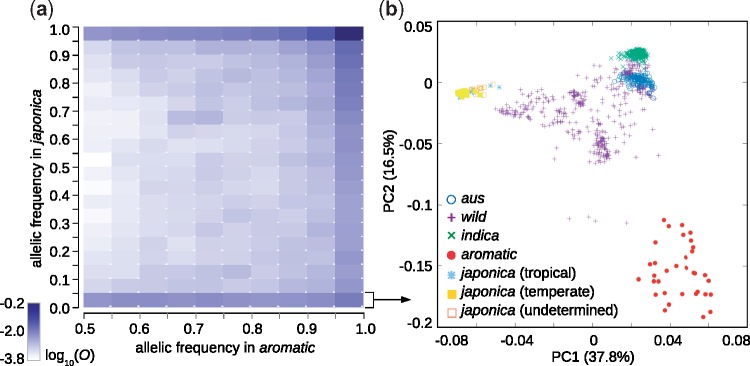
—Non-*japonica* variants present in the *aromatic* group. (*a*) Joint allele frequency spectrum summarizing frequency distribution of major *aromatic* alleles and their occurrence in *japonica*. Logarithmic transformation was used for the observed frequencies. The largest fraction of the examined sites is invariant in the two groups (the top-right field); however, 100,016 major *aromatic* alleles are rare or absent in *japonica* (the bottom row). (*b*) PCA constructed from the 100,016 sites identified above.

In order to check whether *aromatic*’s variation is merely a subset of *japonica*’s variation, or instead contains variants that are absent from *japonica*, we summarized the major *aromatic* alleles (allelic frequency >0.5) at 1,807,643 sites on a joint allele frequency spectrum (fig. 3*a*). The spectrum shows that a substantial fraction of variants common in *aromatic* are rare or absent in *japonica*. Specifically, 100,016 of the major *aromatic* alleles have ≤0.05 frequency in *japonica*, and 58,168 of those can be considered absent in *japonica* (frequency <0.01). Subsequent PCA computed from the 100,016 sites revealed that *aromatic* is more closely associated with *aus*, *indica*, and some wild accessions when this “non-*japonica*” fraction of the genome is considered. The *aromatic* cluster partially overlaps with *aus*, *indica*, and wild rice along the first PC, and with a few wild accessions along the second PC (fig. 3*b*).

### Origin of the Non-Japonica Fraction of the Aromatic Gene Pool

We found that the majority of the 58,168 major *aromatic* alleles that are absent in *japonica* can be found in other rice groups at >0.05 frequencies. Specifically, 55.4%, 63.0%, and 86.8% of these alleles are found in *indica*, *aus*, and wild rice, respectively. This indicates that the majority of the non-*japonica* fraction of the *aromatic* gene pool comprises variants that did not emerge in *aromatic* postdomestication, but have a specific ancestor instead. We searched for this specific ancestor by complementing two lines of evidence: 1) per-individual distribution of the major *aromatic* alleles that are absent in *japonica* and 2) comparison of chloroplast haplotypes.

First, we extracted the 58,168 sites from the diversity matrix, and for each wild, *indica*, *aus*, and *japonica* individual we calculated the proportion of sites that match the *aromatic* major allele (supplementary table S1, Supplementary Material online). This effectively measures similarity of those individuals to *aromatic* within the selected genomic fraction. We identified a group of accessions that carry the non-*japonica aromatic* variants in distinctly higher proportions (supplementary fig. S6*a*, Supplementary Material online), consisting of 90 *aus* and 62 wild accessions. Within these accessions mostly coming from the Indian subcontinent, 89.9% of the 58,168 *aromatic* non-*japonica* alleles can be found. Analogically, we identified wild subpopulations distinctly similar to *aus* and *indica* (supplementary fig. S6*b* and *c*, Supplementary Material online) and found that the subpopulations associated with *aromatic* and *aus* have a strong overlap.

**Fig. 4.**
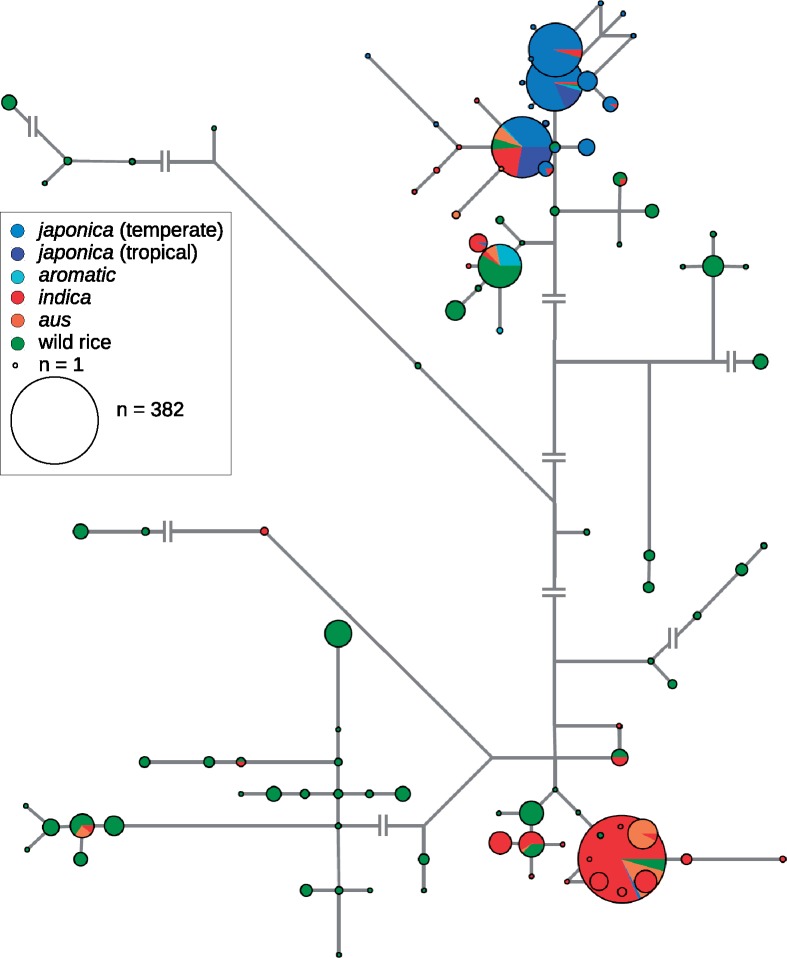
—Median-joining network constructed from the complete chloroplast genome sequences of 1,608 rice accessions. Each node represents a distinct haplotype and is proportional to the number of accessions contained. In most cases, edge lengths are proportional to the number of polymorphisms separating the nodes; otherwise, shortening of edges is indicated. Haplotypes are designated on supplementary fig. S7 and assigned to accessions in supplementary table S2 (Supplementary Material online).

**Fig. 5.**
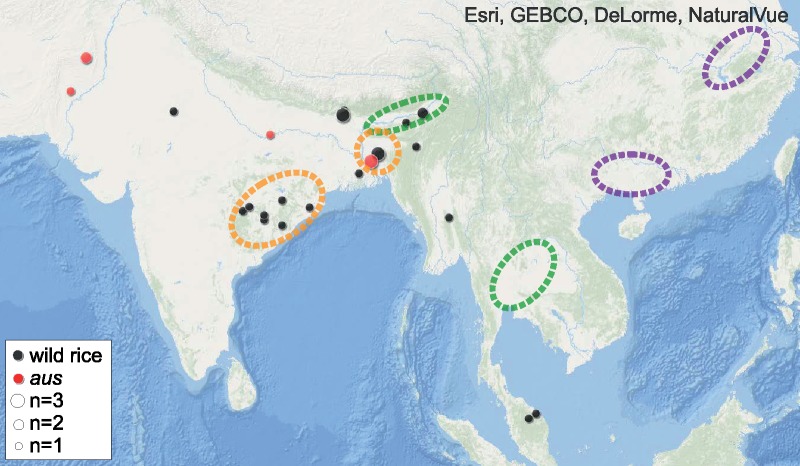
—Extant geographic distribution of the accessions identified as likely ancestors of the non-*japonica* fraction of the *aromatic* genome. The accessions shown as black and red dots are highly similar to *aromatic* in respect to possession of the alleles that are major in *aromatic* but absent in *japonica*, and simultaneously carry the A04 chloroplast haplotype typical for most *aromatic* accessions. Probable areas of *japonica*, *indica* and *aus* domestications (11) are indicated with purple, green and orange dotted ellipses, respectively.

Reconstruction of the complete chloroplast genomes revealed that six (17.6%) *aromatic* individuals have chloroplast haplotypes that are very common in *japonica* (haplotypes A01 and A02; fig. 4 and supplementary fig. S7 and table S2, Supplementary Material online). However, the other 28 *aromatic* individuals (82.4%) carry chloroplast haplotypes A04 or A09 that are not found in *japonica*, and are not derived from the *japonica* haplotypes. This observation provides further evidence for hybrid origin of *aromatic*. Apart from the *aromatic* group, the A04 haplotype is also found in 55 wild, 8 *aus*, and 3 *indica* accessions. In relative proportions per group, the A04 haplotype is common in wild rice (in 12% of the wild accessions), can also be found in *aus* (6.5%), but is very rare in *indica* (0.6%). This leads us to conclude that most of the *aromatic* individuals inherited their chloroplast genomes (hence the cytoplasm) from the non-*japonica* ancestor, which was most likely a specific wild subpopulation or a member of the *aus* group. Therefore, we searched for the A04 haplotype among the accessions that are distinctly similar to the non-*japonica aromatic* fraction. We found the A04 haplotype in seven *aus* and 23 wild accessions, with a geographic distribution centred on the Indian subcontinent (fig. 5). We conclude that these accessions represent the extant populations derived from the most probable non-*japonica* ancestors of the *aromatic* group.

### Local Ancestry Inference

Identification of the extant lineages representing the source populations of the non-*japonica* component of *aromatic* allows per-individual inference of local ancestry across the entire genome, using modeling tools such as HAPMIX (Price et al. 2009) and Loter (Dias-Alves et al. 2018). We first tested the ability of Loter to correctly reconstruct local ancestry of in silico recombinants created from the putative source populations of the *aromatic* group. We found that Loter is able to capture the overall ancestry pattern, albeit with fine-scale errors that are often consistent across samples (supplementary fig. S8, Supplementary Material online). These in silico reconstructions revealed a systematic bias leading to underestimation of the non-*japonica* ancestry by a factor of 1.22, likely resulting from the differences in the proportions of missing data between the source populations.

**Fig. 6.**
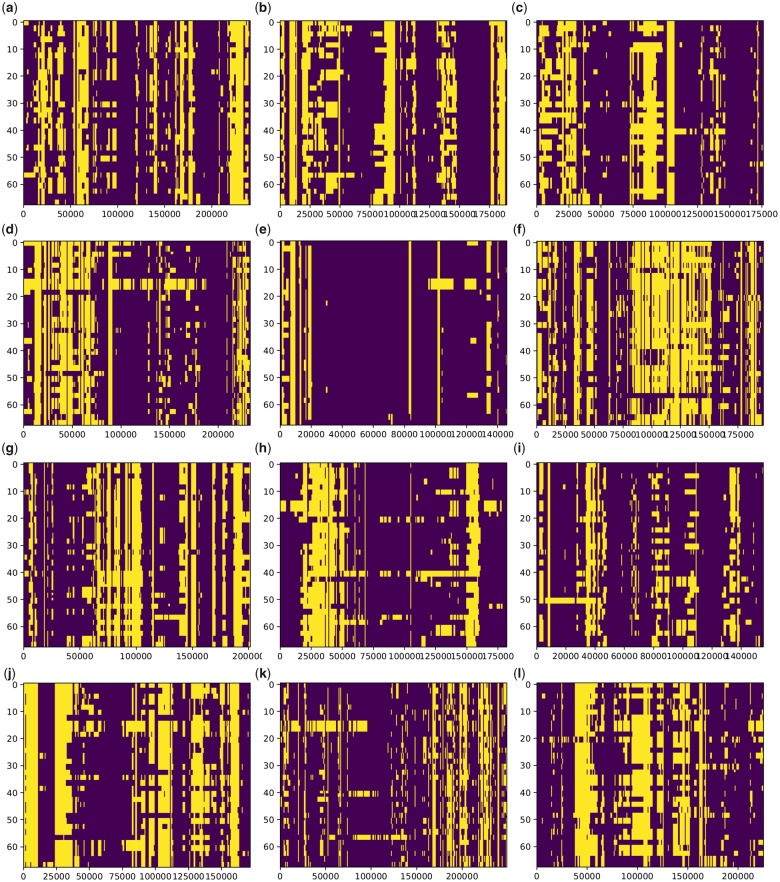
—Local ancestry inference for 34 *aromatic* individuals. Each row corresponds to a single chromosome, that is, each individual is represented by two rows. Columns represent polymorphic sites; the *x*-axis indicates the number of sites examined (not their physical location). Purple—*japonica* ancestry; yellow—non-*japonica* (Indian) ancestry; (*a–l*) chromosomes 1–12.

The local ancestry inference of the *aromatic* individuals indicates that a significant portion of their genomes originated from the non-*japonica* source (fig. 6). Following the bias correction, the results suggest that on an average, 36% of the *aromatic* genome can be traced to this source, the proportion ranging from 29% to 47% among individuals. The highly consistent pattern of the local ancestry across individuals indicates that the whole *aromatic* group stems from a single admixture event, although the original genomic footprint has in some lineages been disrupted by subsequent recombinations.

Inspection of the local ancestry at the loci related to the domestication phenotype revealed that most of the examined genes in the *aromatic* group have either *japonica* or mixed origins (supplementary table S3, Supplementary Material online). Among the genes often considered crucial for the basic domestication characteristics, the *Rc* gene (responsible for white pericarp; Sweeney et al. 2006 and reduction of seed dormancy in cultivated rice Gu et al. 2011), the *Prog1* gene (erect growth; Tan et al. 2008) and the *LABA1* gene (short and barbless awns; Hua et al. 2015) were uniformly inherited from the *japonica* ancestor. Several genes controlling grain size, shape, and yield also have complete *japonica* ancestry. Importantly, most but not all *aromatic* accessions carry *japonica*-like haplotypes of the *S5* gene involved in the reproductive separation of *indica* and *japonica* (Chen J et al. 2008), which is the likely cause of the limited cross-compatibility between *aromatic* and *indica*. The ancestry of the *Badh2* gene (fragrance; Bradbury et al. 2008; Chen S et al. 2008) is traced to the *japonica* ancestor in all fragrant members of the *aromatic* group, in accordance to the conclusions by Kovach et al. (2009). Interestingly, in our set of 34 *aromatic* representatives, only eight accessions carry the *badh2.1* deletion, and other mutations previously reported as interrupting the reading frame of the *Badh2* gene (Kovach et al. 2009) were not found. Out of the 76 accessions classified as *aromatic* in the full 3k RGP data set, only 26 carry the fragrance-causing *badh2.1* allele, highlighting that fragrance is not the defining feature of the *aromatic* group.

Genes influencing starch synthesis, amylose content and gelatinization temperature were inherited from both source populations. The *Sh4* gene (nonshattering ear; Li et al. 2006) also shows mixed ancestry in *aromatic*, with two thirds of the examined accessions inheriting the locus from the non-*japonica* ancestor. Only one of the examined genes has a higher level of non-*japonica* ancestry—the *PCR1* gene influencing grain weight and Zn^2+^ accumulation (Song et al. 2015) is uniformly inherited from this ancestor.

## Discussion

The origins of *aromatic* rice have been uncertain. Its morphology and distribution on the Indian subcontinent led to an initial association with *indica* (Glaszmann 1986), but *aromatic* has distinctive features (phenol reaction, opaque kernel appearance, intermediate amylose content and medium gel consistency) that prompted suggestions that it might be intermediate between *indica* and *japonica* (Ahuja et al. 1995; Bhattacharjee et al. 2002). Later studies of genetic diversity revealed that *aromatic* is closer to and possibly a subgroup of *japonica* (Garris et al. 2005; Kovach et al. 2009; Zhao et al. 2011; 3,000 Rice Genomes Project 2014). As *japonica* clearly originated in East Asia (Huang et al. 2012; Civáň et al. 2015; Civáň and Brown 2018), but *aromatic* is not found in China, it appears that *aromatic* emerged after *japonica* cultivation reached the Indian subcontinent, possibly following hybridization between *japonica* and a local variety of rice. Analysis of genomic regions with reduced diversity in all groups of cultivated rice supported this scenario, although only a small sample of *aromatic* accessions was examined (Civáň et al. 2015). This study revealed that among such regions, which arguably were targeted by artificial selection, about a quarter of *aromatic* haplotypes are associated with *japonica*, another quarter are unique, while about one-fifth are associated with *aus*, indicating that *aromatic* could have emerged from hybridization between *japonica* and *aus*.

To make a more comprehensive analysis of *aromatic* origins we merged genomic data for cultivated and wild rice (Huang et al. 2012; 3,000 Rice Genomes Project 2014), and analyzed this data set with a variety of population genomics tools. PCA of population diversity placed the *aromatic* accessions between *japonica* and the other cultivated groups along the top two axes of variation (fig. 2*a*), while *aromatic* was clearly separated from all other rice along the third axis of variation (fig. 2*b*), in agreement with previous observations (Zhao et al. 2011). Based on the PCA, it is difficult to conclude whether the position of the *aromatic* cluster is due to genetic interaction between two groups, or due to the limitations of 2D projections of a multidimensional space. Genome-wide scans of nucleotide diversity and pairwise *F*_ST_ confirmed that parts of the *aromatic* and *japonica* genomes are almost identical (supplementary fig. S3, Supplementary Material online), indicating common ancestry. Nonetheless, the *F*_ST_ scans and the joint allele frequency spectrum (fig. 3) showed that a substantial fraction of *aromatic* genome is dissimilar from *japonica* and of unknown provenance.

The maternal lineage record of the chloroplast genomes has previously demonstrated deep divergence between *indica* and *japonica* (Kawakami et al. 2007; Kumagai et al. 2016; Tong et al. 2016). Our results show that most *aromatic* accessions carry a chloroplast lineage that is not present in or derived from *japonica*, but is relatively frequent in other rice groups (supplementary table S2 and fig. S7, Supplementary Material online), strengthening the hypothesis that *japonica* is not the sole ancestor of *aromatic*. Further analyses of the nuclear variation revealed that the major *aromatic* alleles that are absent in *japonica* can often be found in *indica* and *aus* (accounting for 55.4% and 63% of such alleles, respectively), but their highest fraction is present in the wild superpopulation (86.8%). After integrating the chloroplast and nuclear diversity data, we identified a group of 30 accessions that best represent the extant lineages of the non-*japonica* ancestor of *aromatic* rice (fig. 5). This group consists of some wild accessions sampled mainly from the Indian subcontinent, together with a few *aus* accessions. Treating this subset of 30 accessions as the non-*japonica* source of the admixed *aromatic* gene pool, per-individual local ancestry suggested that 7 [29–47% (mean 36%)] of the *aromatic* genome is derived from the non-*japonica* source. Local ancestry at selected domestication loci (supplementary table S3, Supplementary Material online) indicates that the non-*japonica* ancestor did not have a domesticated phenotype, since *aromatic* accessions inherited most of the crucial haplotypes from *japonica*. Interestingly, the *Sh4* gene is an exception from this pattern, as it is assigned non-*japonica* ancestry in the majority of the *aromatic* accessions. This result is consistent with the observation of multiple haplotypes at the domestication loci in *O. sativa* (Wang et al. 2018) and suggests that the recessive *sh4* allele was already present in the non-*japonica* ancestor. It is possible that the seed shattering locus was not under selection during the admixture event and became fixed later (Ray and Chakraborty 2018).

Our population genomics approach therefore shows that *aromatic* rice arose from hybridization between *japonica* and a wild rice, related to *aus*, that was local to the Indian subcontinent. A recent preprint by Choi et al. (2018) reported a phylogenomic approach to resolve the origin of the *aromatic* cultivar Basmati 334, by comparison with a selection of high-quality *Oryza* sp. genomes. By counting alternative topologies of gene trees across the genome in a three-species phylogeny, they found that 51.3% of the informative gene trees group Basmati 334 with cv. Nipponbare (*japonica*), while 39.7% group Basmati 334 with an *O. rufipogon* accession. When the three-species phylogenetic reconstruction involves Basmati 334 with the representatives of *aus* (cv. N22), and *indica* (cv. R498), 53.4% of the informative gene trees group *aus* with *indica*, while 26.5% group *aus* with Basmati 334. The authors concluded that this indicates admixture events between Basmati 334, *aus* and *O. rufipogon*. Although this phylogenomic approach, involving only a handful of genomes, cannot provide detailed insights into the demography and geography of the proposed admixture events, the results of Choi et al. (2018) are consistent with our findings.

The results that we report, together with our previous work on the population origins of *indica* and *aus* (Civáň et al. 2015; Civáň and Brown 2018) offer a new perspective on the genetic identity of the early rice cultivated on the Indian subcontinent. According to the “proto-*indica* hypothesis” (Fuller et al. 2010; Fuller 2011), early rice exploitation on the Indian subcontinent was based on predomesticated *indica* grown in the Ganges region, with the fully domesticated *indica* emerging as a result of hybridization with domesticated *japonica*, following the spread of the latter from East Asia (Sang and Ge 2007; Huang et al. 2012; Choi et al. 2017). However, the hypothesis that the native Indian rice was *indica*-like is inconsistent with recent results showing that *indica*-specific variants are usually found in wild populations from Thailand and the Brahmaputra valley, while the *aus* group is more similar to the central Indian wild population (Civáň et al. 2015; Civáň and Brown 2018) (supplementary fig. S6*c*, Supplementary Material online). Evidence of *japonica* introgressions in the *indica* genome is also ambiguous (Civáň and Brown 2018; Wang et al. 2018). Importantly, archaeobotanical identification of *indica* relies on the grain length/width ratio (Castillo et al. 2016), which is a poor proxy for classification of modern germplasm (Morishima and Oka 1981). Moreover, archaeobotanists do not distinguish *aus* from *indica* (Fuller et al. 2016), and the differentiation of *aus* and the morphologically diverse *aromatic* group (Islam et al. 2016; Lahkar and Tanti 2017) from *indica* and *japonica* is beyond the discriminating power of rice morphometrics. These archaeobotanical limitations lend further uncertainty in the proto-*indica* hypothesis.

The results presented here and previously (Civáň et al. 2015; Civáň and Brown 2018) indicate that the wild rice populations south of the Himalayas were ancestral to *aus* and the non-*japonica* fraction of *aromatic*, but not to *indica*. We conclude that the domestication phenotype of *aus* arose independently on the Indian subcontinent (Civáň and Brown 2018), followed by a later influx of the *japonica* population some 4,000 years ago (Petrie et al. 2016; Bates et al. 2017), the latter accompanied by the emergence of *aromatic* through hybridization with wild rice along the foothills of the Himalayas. *Japonica* also interacted with *aus*, though to a much lesser extent (e.g., donating a large portion of the chromosome 7) (Civáň and Brown 2018) and the diversity of cultivated rice in the region was further enriched by the spread of *indica*, which entered the Indian plains either from the Brahmaputra valley or from Southeast Asia. Each these groups largely maintained their genetic integrity during the following millennia of cultivation, probably due to cross-compatibility barriers and differences in agroecological settings. 

## Supplementary Material

Supplementary data are available at *Genome Biology and Evolution* online.

## Supplementary Material

Supplementary Data

## Notes

**Data deposition:** The merged *O. sativa–O. rufipogon* vcf file has been deposited on Mendeley Data (doi: 10.17632/74dbx8bmp9.1). The reconstructed chloroplast genomes with total quality scores are available from zenodo (doi: 10.5281/zenodo.61977).

## References

[evz039-B1] 3,000 Rice Genomes Project. 2014 The 3,000 rice genomes project. GigaScience 3:7. 24872877 10.1186/2047-217X-3-7 PMC4035669

[evz039-B2] Ahuja SC, Panwar DVS, Ahuja U, Gupta KR. 1995 In: Bansal RP, editor. Basmati rice: the scented pearl. Hisar: CCS Haryana Agricultural University p. 1–63.

[evz039-B3] Bandelt HJ, Forster P, Rőhl A. 1999 Median-joining networks for inferring intraspecific phylogenies. Mol Biol Evol. 16(1):37–48. 10331250 10.1093/oxfordjournals.molbev.a026036

[evz039-B4] Bates J, Petrie CA, Singh RN. 2017 Approaching rice domestication in South Asia: new evidence from Indus settlements in northern India. J Archaeol Sci. 78:193–201. 10.1016/j.jas.2016.04.018 PMC7773629 33414573

[evz039-B5] Bhattacharjee P, Singhal RS, Kulkarni PR. 2002 Basmati rice: a review. Int J Food Sci Technol. 37(1):1–12.

[evz039-B6] Bolger AM, Lohse M, Usadel B. 2014 Trimmomatic: a flexible trimmer for Illumina Sequence Data. Bioinformatics 30(15):2114–2120. 24695404 10.1093/bioinformatics/btu170 PMC4103590

[evz039-B7] Bradbury L, Gillies S, Brushett D, Waters D, Henry R. 2008 Inactivation of an aminoaldehyde dehydrogenase is responsible for fragrance in rice. Plant Mol Biol. 68(4–5):439–449. 18704694 10.1007/s11103-008-9381-x

[evz039-B8] Browning SR, Browning BL. 2007 Rapid and accurate haplotype phasing and missing data inference for whole genome association studies by use of localized haplotype clustering. Am J Hum Genet. 81(5):1084–1097. 17924348 10.1086/521987 PMC2265661

[evz039-B9] Bryant D, Moulton V. 2004 Neighbor-Net: an agglomerative method for the construction of phylogenetic networks. Mol Biol Evol. 21(2):255–265. 14660700 10.1093/molbev/msh018

[evz039-B10] Castillo CC, et al 2016 Archaeogenetic study of prehistoric rice remains from Thailand and India: evidence of early *japonica* in South and Southeast Asia. Archaeol Anthropol Sci. 8(3):523–543.

[evz039-B11] Chang CC, et al 2015 Second-generation PLINK: rising to the challenge of larger and richer datasets. GigaScience 4:7. 25722852 10.1186/s13742-015-0047-8 PMC4342193

[evz039-B12] Chen J, et al 2008 A triallelic system of *S5* is a major regulator of the reproductive barrier and compatibility of *indica-japonica* hybrids in rice. Proc Natl Acad Sci U S A. 105(32):11436–11441. 18678896 10.1073/pnas.0804761105 PMC2516230

[evz039-B13] Chen S, et al 2008 *Badh2*, encoding betaine aldehyde dehydrogenase, inhibits the biosynthesis of 2-acetyl-1-pyrroline, a major component in rice fragrance. Plant Cell 20(7):1850–1861. 18599581 10.1105/tpc.108.058917 PMC2518245

[evz039-B14] Choi JY, et al 2017 The rice paradox: multiple origins but single domestication in Asian rice. Mol Biol Evol. 34(4):969–979. 28087768 10.1093/molbev/msx049 PMC5400379

[evz039-B15] Choi JY, Groen S, Zaaijer S, Purugganan M. 2018 Nanopore sequence-based genome assembly of the basmati rice. Preprint at 10.1101/396515. PMC7001208 32019604

[evz039-B16] Civáň P, Brown TA. 2016 Diversity patterns across 1,800 chloroplast genomes of wild (*Oryza rufipogon* Griff.) and cultivated rice (*O. sativa* L.). Preprint at 10.1101/094482.

[evz039-B17] Civáň P, Brown TA. 2017 Origin of rice (*Oryza sativa* L.) domestication genes. Genet Resour Crop Evol. 6:1125–1132. 10.1007/s10722-017-0518-0 PMC5498617 28736485

[evz039-B18] Civáň P, Brown TA. 2018 Role of genetic introgression during the evolution of cultivated rice (*Oryza sativa* L.). BMC Evol Biol. 18(1):57. 29688851 10.1186/s12862-018-1180-7 PMC5913815

[evz039-B19] Civáň P, Craig H, Cox CJ, Brown TA. 2015 Three geographically separate domestications of Asian rice. Nat Plants. 1:15164. 27251535 10.1038/nplants.2015.164 PMC4900444

[evz039-B20] Danecek P, et al 2011 The variant call format and VCFtools. Bioinformatics 27(15):2156–2158. 21653522 10.1093/bioinformatics/btr330 PMC3137218

[evz039-B21] Dias-Alves T, Mairal J, Blum MGB. 2018 Loter: a software package to infer local ancestry for a wide range of species. Mol Biol Evol. 10.1093/molbev/msy126. PMC6107063 29931083

[evz039-B22] Engle LM, Chang TT, Ramirez DA. 1969 The cytogenetics of sterility in F1 hybrids of *indica×indica and indica×javanica* varieties of rice (*Oryza sativa* L.). Philipp Agric. 53:289–307.

[evz039-B23] Fuller DQ. 2011 Pathways to Asian civilizations: tracing the origins and spread of rice and rice cultures. Rice 4(3–4):78–92.

[evz039-B24] Fuller DQ, et al 2010 Consilience of genetics and archaeobotany in the entangled history of rice. Archaeol Anthropol Sci. 2(2):115–131.

[evz039-B25] Fuller DQ, Weisskopf AR, Castillo CC. 2016 Pathways of rice diversification across Asia. Archaeol Int. 19:84–96.

[evz039-B26] Garris AJ, Tai TH, Coburn J, Kresovich S, McCouch S. 2005 Genetic structure and diversity in *Oryza sativa* L. Genetics 169(3):1631–1638. 15654106 10.1534/genetics.104.035642 PMC1449546

[evz039-B27] Glaszmann JC. 1986 A varietal classification of Asian cultivated rice (*Oryza sativa* L.) based on isozyme polymorphism In: Banta SJ, editor. Rice genetics I, Part 2, Manila: IRRI p. 83–90.

[evz039-B28] Glaszmann JC. 1987 Isozymes and classification of Asian rice varieties. Theor Appl Genet. 74(1):21–30. 24241451 10.1007/BF00290078

[evz039-B29] Gu X-Y, et al 2011 Association between seed dormancy and pericarp color is controlled by a pleiotropic gene that regulates abscisic acid and flavonoid synthesis in weedy red rice. Genetics 189(4):1515–1524. 21954164 10.1534/genetics.111.131169 PMC3241415

[evz039-B30] Hua L, et al 2015 *LABA1*, a domestication gene associated with long, barbed awns in wild rice. Plant Cell 27(7):1875–1888. 26082172 10.1105/tpc.15.00260 PMC4531357

[evz039-B31] Huang X, et al 2012 A map of rice genome variation reveals the origin of cultivated rice. Nature 490(7421):497–503. 23034647 10.1038/nature11532 PMC7518720

[evz039-B32] Ishii T, et al 2013 *OsLG1* regulates a closed panicle trait in domesticated rice. Nat Genet. 45(4):462–465. 23435087 10.1038/ng.2567

[evz039-B33] Islam MZ, et al 2016 Variability assessment of aromatic and fine rice germplasm in Bangladesh based on quantitative traits. ScientificWorldJournal 2016:1. 10.1155/2016/2796720 PMC4830757 27127800

[evz039-B34] Kawakami S, et al 2007 Genetic variation in the chloroplast genome suggests multiple domestications of cultivated Asian rice (*Oryza sativa* L.). Genome 50(2):180–187. 17546083 10.1139/g06-139

[evz039-B35] Khush GS. 2000 Taxonomy and origin of rice In: Singh RK, Singh US, Khush GS, editors. Aromatic rices. New Delhi: Oxford & IBH Publishing p. 5–13.

[evz039-B36] Kovach MJ, Calingacion MN, Fitzgerald MA, McCouch SR. 2009 The origin and evolution of fragrance in rice (*Oryza sativa* L.). Proc Natl Acad Sci U S A. 106(34):14444–14449. 19706531 10.1073/pnas.0904077106 PMC2732888

[evz039-B37] Kumagai M, et al 2016 Rice varieties in archaic east Asia: reduction of its diversity from past to present times. Mol Biol Evol. 33(10):2496–2505. 27461246 10.1093/molbev/msw142

[evz039-B38] Lahkar L, Tanti B. 2017 Study of morphological diversity of traditional aromatic rice landrace (*Oryza sativa* L.) collected from Assam, India. Ann Plant Sci. 6(12):1855–1861.

[evz039-B39] Li C, Zhou A, Sang T. 2006 Rice domestication by reducing shattering. Science 311(5769):1936–1939. 16527928 10.1126/science.1123604

[evz039-B40] Li H, Durbin R. 2009 Fast and accurate short read alignment with Burrows-Wheeler transform. Bioinformatics 25(14):1754–1760. 19451168 10.1093/bioinformatics/btp324 PMC2705234

[evz039-B41] McKenna A, et al 2010 The Genome Analysis Toolkit: a MapReduce framework for analysing next-generation DNA sequencing data. Genome Res. 20(9):1297–1303. 20644199 10.1101/gr.107524.110 PMC2928508

[evz039-B42] Monat C, et al 2015 TOGGLE: toolbox for generic NGS analyses. BMC Bioinformatics 16:374. 26552596 10.1186/s12859-015-0795-6 PMC4640241

[evz039-B43] Morishima H, Oka HI. 1981 Phylogenetic differentiation of cultivated rice, 22. Numerical evaluation of the Indica-Japonica differentiation. Jpn J Breed. 31(4):402–413.

[evz039-B44] Oka HI. 1988 Indica-Japonica differentiation of rice cultivars. In: Oka HI, editor. Origin of cultivated rice. Tokyo: Japan Scientific Societies Press; Amsterdam: Elsevier Science Publishers. p. 141–179.

[evz039-B45] Patterson N, Price AL, Reich D. 2006 Population structure and eigenanalysis. PLoS Genet. 2(12):e190. 17194218 10.1371/journal.pgen.0020190 PMC1713260

[evz039-B46] Petrie CA, Bates J, Higham T, Singh RN. 2016 Feeding ancient cities in South Asia: dating the adoption of rice, millet and tropical pulses in the Indus civilisation. Antiquity 90(354):1489–1504.

[evz039-B47] Price AL, et al 2009 Sensitive detection of chromosomal segments of distinct ancestry in admixed populations. PLoS Genet. 5(6):e1000519. 19543370 10.1371/journal.pgen.1000519 PMC2689842

[evz039-B48] Ray A, Chakraborty D. 2018 Shattering or not shattering: that is the question in domestication of rice (*Oryza sativa* L.). Genet Res Crop Evol. 65(2):391–395.

[evz039-B49] Sang T, Ge S. 2007 Genetics and phylogenetics of rice domestication. Curr Opin Genet Dev. 17(6):533–538. 17988855 10.1016/j.gde.2007.09.005

[evz039-B50] Singh RK, et al 2000 Small and medium grained aromatic rices of India In: Singh RK, Singh US, Khush GS, editors. Aromatic rices. New Delhi: Oxford & IBH Publishing p. 155–177.

[evz039-B51] Song W-Y, et al 2015 Rice *PCR1* influences grain weight and Zn accumulation in grains. Plant Cell Environ. 38(11):2327–2339. 25854544 10.1111/pce.12553

[evz039-B52] Sweeney MT, Thomson MJ, Pfeil BE, McCouch S. 2006 Caught red-handed: *rc* encodes a basic helix-loop-helix protein conditioning red pericarp in rice. Plant Cell 18(2):283–294. 16399804 10.1105/tpc.105.038430 PMC1356539

[evz039-B53] Tan L, et al 2008 Control of a key transition from prostrate to erect growth in rice domestication. Nat Genet. 40(11):1360–1364. 18820699 10.1038/ng.197

[evz039-B54] Tong W, Kim T-S, Park Y-J. 2016 Rice chloroplast genome variation architecture and phylogenetic dissection in diverse *Oryza* species assessed by whole-genome resequencing. Rice 9(1):57. 27757948 10.1186/s12284-016-0129-y PMC5069220

[evz039-B55] Turland NJ, et al 2018 International code of nomenclature for algae, fungi, and plants (Shenzhen Code) In: Turland NJ, et al, editors. Regnum Vegetabile 159. Glashütten: Koeltz Botanical Books p. 1–254.

[evz039-B56] Virmani SS, Chaudhary RC, Khush GS. 1981 Current outlook on hybrid rice. Oryza 18:67–84.

[evz039-B57] Wang W, et al 2018 Genomic variation in 3,010 diverse accessions of Asian cultivated rice. Nature 557(7703):43–49. 29695866 10.1038/s41586-018-0063-9 PMC6784863

[evz039-B58] Zhao K, et al 2011 Genome-wide association mapping reveals a rich genetic architecture of complex traits in *Oryza sativa*. Nat Commun. 2:467. 21915109 10.1038/ncomms1467 PMC3195253

