# Supplementary material for: Origin of the Aromatic Group of Cultivated Rice (Oryza sativa L.) Traced to the Indian Subcontinent
Source: Genome Biol Evol. 2019 Feb 21;11(3):832–43. doi: 10.1093/gbe/evz039 (PMC6427689; doi:10.1093/gbe/evz039)
Supplement: Supplementary Data [file evz039_supp.zip › Civan_2018_SI_GBE_v2.doc]

Supplementary Information for

Origin of the *aromatic* group of cultivated rice (*Oryza sativa* L.) traced to the Indian subcontinent

Peter Civáň, Sajid Ali, Riza Batista-Navarro, Konstantina Drosou, Chioma Ihejieto, Debarati Chakraborty, Avik Ray, Pierre Gladieux, Terence A. Brown

Peter Civáň

Email: peter.civan@inra.fr

**This PDF file includes:**

supplementary figures S1 to S8

**Other supplementary materials for this manuscript include the following:**

supplementary tables S1 to S3

**Fig. S1. PCA of the LD pruned 404k CoreSNP dataset.** Accessions were assigned to populations according to the information from the Sequence Read Archive (ERP005654). Although clusters of *indica*, *aus*, *japonica* and *aromatic* accessions can be identified, a large number of accessions are misclassified or take ambiguous positions.

**Fig. S2. SNP density in the merged *sativa/rufipogon* dataset.** The density was calculated in 100 kb genomic windows.

**Fig. S3**. **Genome-wide diversity**

**scans relevant for the origin of *aromatic*.** Trend lines were calculated as moving averages of ten data points. Locations of genes with relevance to domestication are indicated with black arrowheads. **a**, nucleotide diversity in 100 kb windows across the *aromatic* genome. **b**, pairwise F_ST_ values calculated for *aromatic* and *japonica* in 100 kb windows. Grey shading indicates examples of genomic regions where low nucleotide diversity in *aromatic* coincides with low pairwise *aromatic-japonica* F_ST_. **c**, pairwise F_ST_ values calculated for *aromatic* and *aus* in 100 kb windows. **d**, pairwise F_ST_ values calculated for *aromatic* and *indica* in 100 kb windows.

**Fig. S4. NeighborNet constructed from the full sample set.** Pink, *aromatic*; blue, *japonica*; red, *indica*; orange, *aus*; green, wild rice. Accessions identified as the non-*japonica* ancestor of *aromatic* have black circle lines.

**Fig. S5. NeighborNet constructed from the cultivated accessions.** Nodes are coloured as above. Edges that separate *japonica+aromatic* from all other samples are highlighted in blue; edges separating *aromatic+aus* from all other accessions are highlighted in orange.

**Fig. S6. Histograms of per-individual proportion of alleles that are absent in *japonica* (allelic frequency <0.01), but are the major variants (allelic frequency >0.5) in *aromatic* (a), *aus* (b) and *indica* (c).** All *japonica* accessions have near-zero proportion of such variants (by definition). **a**, occurrence of 58,168 variants found to be absent in *japonica* but major in *aromatic* was examined in each *japonica*, *aus*, *indica* and wild individual. A distinct group of individuals frequently carrying such variants is delineated with a rectangle comprising 90 *aus* and 62 wild accessions. Countries and states of origin for those 62 wild accessions are summarized on the right panel map. **b**, occurrence of 158,511 variants found to be absent in *japonica* but major in *aus* was examined in each *japonica*, *indica*, *aromatic* and wild individual. The rectangle comprises 52 exclusively wild accessions with their geographic origins summarized on the right panel. **c**, occurrence of 117,649 variants found to be absent in *japonica* but major in *indica* was examined in each *japonica*, *aus*, *aromatic* and wild individual. The rectangle comprises 46 exclusively wild accessions with their geographic origins summarized on the right panel.

**Fig. S7. Median-joining network constructed from the complete chloroplast genome sequences of 1,608 rice accessions.** Six haplogroups (A–F) and 107 haplotypes are labelled. Each node represents a distinct haplotype identified by the haplogroup letter and the node number. The haplotypes are numbered according to their ordered frequency, with haplotype 1 being the most frequent. Edge lengths are proportional to the number of polymorphisms separating the nodes, except the edge between the haplotypes o3 and o7 (shortened); the node sizes are proportional to the number of samples. This is an unrooted network, however the root is most likely placed among the “o” (outgroup) haplotypes. This branch includes African *Oryza barthii* and *Oryza glaberrima* (o4), *Oryza longistaminata* (o7), *Oryza meridionalis* (o1-o3, o6) as well as *O. rufipogon* accessions sampled in Australia and Indonesia. Additional details given in Supplementary Table 2.

**Fig. S8. Local ancestry reconstruction of *in silico* recombinants.** Each row corresponds to a single haplotype, i.e., each recombinant is represented by two rows. Columns represent SNP positions; the *x*-axis indicates the number of sites examined (not their physical location). Purple – *japonica* ancestry; yellow – non-*japonica* (Indian) ancestry; **a–l**, chromosomes 1-12. The correct ancestry for each individual is two equally-sized ancestry blocks, one *japonica* (occupies the first half of each chromosome for the haplotypes 0-13 and the second half for the haplotypes 14-27) and the other non-*japonica* (Indian).
